# 阻塞性睡眠呼吸暂停合并肺癌的研究进展

**DOI:** 10.3779/j.issn.1009-3419.2026.101.06

**Published:** 2026-03-20

**Authors:** He CAO, Shanshan YANG, Wenjia SUN, Jianya ZHOU

**Affiliations:** ^1^310000 杭州，浙江大学医学院附属第一医院呼吸与危重症医学科; ^1^Department of Pulmonary and Critical Care Medicine, The First Affiliated Hospital, Zhejiang University School of Medicine, Hangzhou 310000, China

**Keywords:** 阻塞性睡眠呼吸暂停, 肺肿瘤, 慢性间歇性低氧, 病理生理机制, 流行病学, Obstructive sleep apnea, Lung neoplasms, Chronic intermittent hypoxia, Pathophysiological mechanism, Epidemiology

## Abstract

阻塞性睡眠呼吸暂停（obstructive sleep apnea, OSA）是临床常见的睡眠障碍性疾病，其典型特征为睡眠过程中反复发生的上气道塌陷与呼吸暂停现象，可引起间歇性低氧、氧化应激及全身炎症反应，并与心血管疾病、认知功能障碍及代谢异常等多系统功能损害密切相关。肺癌作为全球肿瘤相关死亡的主要原因之一，严重威胁患者的生命健康。近年来研究发现OSA与肺癌具有吸烟、高龄及肥胖等共同危险因素，且二者在病理生理机制上存在一定交叉性，提示OSA可能参与肺癌的发生发展过程。然而，目前关于OSA合并肺癌的流行病学证据仍不一致，其潜在分子机制及临床干预价值尚未得到系统阐明，相关研究仍存在一定不足。故本文拟从OSA合并肺癌的流行病学现状、可能的病理生理机制以及治疗策略等方面进行综述，以期为后续临床治疗决策以及机制研究提供理论依据及参考。

## Abstract (en)

Obstructive sleep apnea (OSA) is a common sleep-related breathing disorder characterized by recurrent upper airway collapse and apnea during sleep, resulting in intermittent hypoxia, oxidative stress, and systemic inflammation, and is closely associated with multisystem dysfunction, including cardiovascular disease, cognitive impairment, and metabolic abnormalities. Lung cancer remains one of the leading causes of cancer-related mortality worldwide and represents a major threat to human health. Recent studies suggest that OSA and lung cancer share several common risk factors, including smoking, older age, and obesity, and exhibit overlapping pathophysiological mechanisms, indicating that OSA may be involved in the initiation and progression of lung cancer. However, the epidemiological evidence regarding the association between OSA and lung cancer remains inconsistent, and the underlying molecular mechanisms as well as the clinical value of potential interventions have yet to be systematically elucidated. This review therefore summarizes the epidemiological characteristics, potential pathophysiological mechanisms, and therapeutic strategies related to the coexistence of OSA and lung cancer, with the aim of providing a theoretical basis for clinical decision-making and future mechanistic studies.

阻塞性睡眠呼吸暂停（obstructive sleep apnea, OSA）是临床常见的睡眠障碍性疾病，其典型病理特征为睡眠过程中反复发生的上气道塌陷与呼吸暂停现象，进而导致慢性间歇性低氧（chronic intermittent hypoxia, CIH）及睡眠片段化。众多研究^[1,2]^已证实OSA表现出显著的年龄与性别差异，并与心血管疾病、认知功能障碍及代谢异常等多系统功能受损密切相关。

肺癌仍是全球恶性肿瘤中发病率和死亡率最高的恶性肿瘤，虽然靶向治疗和免疫治疗已显著改善了肺癌患者预后，但是现如今肺癌治疗仍存在诸多未满足的临床需求。近年来多项研究已证实OSA可以通过多种特定机制促进恶性肿瘤的发生与发展，在肺癌中尤为突出。多项临床研究^[3,4]^表明OSA患者群体具有更高的肺癌发病率和死亡率。肺癌作为目前全球范围内肿瘤负担的重要组成部分，其流行病学特征仍亟需深入研究。近期一项前瞻性临床研究^[5]^发现约半数新诊断的肺癌患者合并中重度OSA，且多数患者同时具备吸烟、高龄和肥胖等高危因素，提示二者之间可能共享相似的病理生理基础，从而增加了疾病共患的可能性。

但是当前关于OSA合并肺癌的研究仍存在较多不足与挑战，因此本文拟从OSA合并肺癌的流行病学现状、病理生理机制以及治疗策略等多角度进行文献综述，以期为后续临床治疗决策以及基础研究提供理论依据及参考。

## 1 OSA合并肺癌的流行病学现状

### 1.1 OSA合并肺癌的发病率

多项临床研究已证实OSA与肺癌发病风险显著相关，这种相关性可能源于OSA特征性的间歇性低氧（intermittent hypoxia, IH）状态，其通过诱导氧化应激、造成DNA损伤及促进全身炎症反应，从而形成促肿瘤生长的微环境。一项韩国的全国性队列研究^[6]^揭示了OSA与肺癌发病风险之间的显著关联性。在调整收入与共患疾病因素后，OSA患者的肺癌发病风险显著升高（HR=1.95, 95%CI: 1.74-2.18），亚组分析显示女性患者（HR=2.14, 95%CI: 1.69-2.70）风险较男性（HR=1.90, 95%CI: 1.67-2.16）更高，尤其在65岁及以上人群中最为突出（HR=2.99, 95%CI: 2.46-3.64）。另一项在西班牙开展的多中心队列研究^[4]^纳入了来自7家教学医院共计4910例患者，平均随访时间为4.5年。研究结果表明夜间低氧血症与癌症发生存在显著相关性，而且OSA严重程度与肺癌发生风险呈明确的剂量-反应关系。以夜间氧饱和度低于90%的时间比例（percent of night time spent with oxygen saturation <90%, TSat_90_）为参照指标，当TSat_90_>12%时，患癌风险为TSat_90_<12%患者群体的2.33倍（95%CI: 1.57-3.46）。进一步分析表明TSat_90_每增加10单位癌症罹患风险增加7%（HR=1.07, 95%CI: 1.02-1.13），这一效应在65岁以下人群中更为显著。一项来自美国的前瞻性研究^[7]^同样证实OSA可使肺癌发病风险增加约50%（HR=1.52, 95%CI: 1.07-2.17），但是该研究中OSA的诊断主要依赖问卷调查结果而非睡眠呼吸监测报告。Kendzerska等^[8]^的研究也表明重度OSA患者肺癌发病风险增加约30%（HR=1.34, 95%CI: 1.00-1.80），且氧合指标为肺癌发生的独立预测因素。一项随访时间长达20余年的巴塞尔顿健康研究^[9]^也得出了类似的结论。另有一项法国的大型研究^[10]^报道了在6年随访时间中夜间低氧血症患者癌症风险更高，尤其在肥胖及老年且未接受规范化OSA治疗的患者群体中最为突出，其中肺癌与乳腺癌的发病风险显著增加。

上述流行病学差异提示OSA对肺癌发生的影响并非单一效应，而是受到遗传背景与环境暴露交互作用的调控。不同人群在缺氧诱导因子1α（hypoxia inducible factor-1α, HIF-1α）通路、炎症因子及氧化应激相关基因中的遗传多态性可能影响其对IH的生物学反应，从而调控OSA相关肿瘤发生风险^[11]^。此外，吸烟、大气污染及职业暴露等外源性因素可进一步放大CIH介导的DNA损伤、慢性炎症及免疫抑制效应，在肺癌等吸烟相关肿瘤中表现出更为显著的风险叠加效应。OSA在不同的肿瘤类型中也呈现出明显的异质性，在乳腺癌、结直肠癌及前列腺癌等癌症中，既往研究^[12]^未观察到一致的风险升高，甚至在部分人群中呈现出负相关或保护性趋势。这种异质性可能与以下机制相关：（1）CIH可在不同组织微环境中诱导差异化的免疫调控效应，在某些肿瘤中可以激活免疫杀伤细胞功能，增强抗肿瘤免疫作用；（2）不同肿瘤对缺氧的适应能力存在差异，部分肿瘤可能对CIH诱导的氧化应激更为敏感，从而抑制其发生发展。OSA的促癌或抑癌作用受到肿瘤缺氧适应机制、宿主免疫状态以及环境暴露等因素的综合影响。相比之下，由于肺癌与吸烟及环境暴露高度相关，其发生发展更易受到OSA所致CIH及慢性炎症的协同促进作用，这也在一定程度上解释了其在人群研究中更为一致的正相关趋势。

肺癌在组织学上分为非小细胞肺癌（non-small cell lung cancer, NSCLC）和小细胞肺癌（small cell lung cancer, SCLC），组织学亚型分析表明其中NSCLC与OSA的关联最为突出。腺癌较少压迫阻塞中央气道，但是易于转移出现胸腔积液等并发症，可影响呼吸节律及胸廓顺应性，从而加重OSA症状。动物实验^[13]^也证实IH状态可以加速肺腺癌生长，这可能与乏氧状态下的肿瘤微环境和促癌基因表达增加相关。鳞癌多为中央型生长，解剖学上可压迫气道加重气流受限，增加OSA风险。同时IH状态可上调趋化因子表达，增强肺癌细胞侵袭性。

与NSCLC相比，SCLC具有高度增殖性和早期广泛转移的特点，其肿瘤进展更多依赖于系统性扩散而非局部气道阻塞效应，因此OSA通过压迫气道加重肿瘤发生发展的作用在SCLC中可能相对有限。SCLC与NSCLC在生物学行为及对缺氧刺激的反应方面存在显著差异性，这可能导致其与OSA的关联机制不同。OSA形成的IH状态可激活HIF-1α通路、促进血管生成及炎症反应，这些机制在NSCLC中已被证实可促进肿瘤进展。SCLC常伴有TP53和RB1基因失活，对缺氧微环境的依赖性可能不同于NSCLC，部分研究提示SCLC细胞系对持续性低氧更为敏感，而对IH的适应机制尚不明确。SCLC还具有明显的神经内分泌特征，而OSA相关的交感神经激活及全身炎症反应可能通过神经内分泌途径间接影响肿瘤进展，但目前缺乏直接证据支持这一假设。Lee等^[14]^的研究观察到根据肿瘤细胞类型、基因突变状态、肿瘤分期及生存情况分层分析，呼吸暂停低通气指数（apnea-hypopnea index, AHI）、氧减指数（oxygen desaturation index, ODI）以及血氧饱和度低于90%的时间比例虽未观察到显著差异，但是SCLC患者在AHI、ODI及TSat_90_方面呈现较高趋势。Liu等^[15]^研究也发现SCLC亚组预后显著差于NSCLC亚组，提示其可能存在不同的低氧适应或免疫逃逸机制。因此，未来有必要在大样本临床队列基础上结合单细胞测序及动物模型等方法系统性探讨IH对SCLC肿瘤微环境、免疫调控及神经内分泌通路的影响，以进一步阐明OSA在不同肺癌组织学亚型中的差异性作用。

### 1.2 OSA合并肺癌的预后

OSA在肺癌患者中发生率显著升高，且临床证据表明其与肺癌患者的不良预后密切相关。一项纳入5427例受试者的多中心研究^[16]^显示OSA严重程度与癌症死亡风险显著升高有关。此外，一项纳入45例肺癌患者的随访研究^[17]^也发现合并OSA患者在1年内的转移复发率为37.5%，死亡率为41.7%，均高于非OSA患者。尽管两组患者在绝对死亡率、复发率及转移率方面差异无统计学意义，但OSA亚组总体疾病进展显著加快，提示OSA可能促进肺癌进展。Huang等^[18]^的研究证实在III-IV期肺癌人群中，重度OSA患者癌症相关死亡率显著高于轻中度患者，约80%的重度OSA患者在3年内死亡，且患者的预后与HIF-1α表达水平显著相关。Li等^[19]^研究亦发现与轻度OSA相比，中重度OSA肺癌患者生存时间显著缩短，进一步支持OSA严重程度是影响肺癌生存的不利因素。上述临床观察与基础研究结果相一致，OSA所特有的IH状态与持续性缺氧不同，通常表现为反复缺氧-复氧循环，可更显著地诱导HIF-1α的稳定表达及转录活性增强，从而在动态氧波动环境中持续驱动肿瘤适应性反应。多重机制在乏氧条件下协同作用最终形成以免疫抑制为特征的肿瘤微环境，促进肿瘤进展及不良预后。

## 2 OSA合并肺癌的可能发病机制

现有流行病学证据支持OSA与肺癌的发病及预后有显著相关性，但是其内在生物学机制仍有待深入阐明。IH、炎症及免疫调节、氧化应激等生物学效应可能构成连接二者之间的关键机制。

### 2.1 IH效应

OSA的核心病理特征为IH，它通过多重机制重塑肿瘤微环境，其中HIF-1α通路的激活发挥关键作用。有研究^[20]^显示乏氧状态诱导的HIF-1α上调与ATAD2表达升高密切相关，该信号轴显著促进肺癌细胞的干性特征。体外实验^[21]^证实在模拟IH条件下H520细胞增殖能力提升66%（P<0.001），而在单纯OSA模拟的IH条件下，H520和H1437细胞增殖分别增加72%（P<0.001）和40%（P=0.043）。动物实验^[22]^表明年轻小鼠暴露于IH后肿瘤生长速度较暴露于正常空气的对照组增加约55%（P=0.007），而在老年小鼠中未观察到类似现象，这提示年龄可能调控IH的促瘤效应。机制研究进一步表明IH可以通过改变年轻小鼠的免疫状态促进肿瘤进展，年轻小鼠经IH处理后肿瘤内肿瘤相关巨噬细胞（tumor-associated macrophages, TAMs）浸润量增加约85%，而老年小鼠未观察到类似改变^[22]^。此外，两类年龄组的调节性T细胞（regulatory T cells, Tregs）数量均呈上升趋势，但老年组增幅明显低于年轻组，该免疫抑制细胞的差异性累积可能导致年龄相关的抗肿瘤免疫应答差异，这可能与长期缺氧诱导的HIF-1α表达上调有关。但是该研究仍存在一定的局限性，IH动物模型仍难以完全模拟人类OSA的复杂病理状态，实验条件下IH的暴露频率、持续时间及缺氧波动模式难以完全模拟临床上OSA患者的缺氧状态，而且小鼠的免疫系统与人类在组成及调控机制上亦存在显著不同。在该研究中对动物年龄的划分较为单一，而在人群中年龄往往伴随多种合并症及治疗因素，这些均可能影响IH的肿瘤生物学效应。现有流行病学证据提示OSA可能与肿瘤侵袭性增强有关，在较年轻患者中更为显著，但是相关结论主要来源于回顾性研究，今后关于IH在不同年龄人群中的促瘤作用及其机制仍有待更多前瞻性临床研究加以验证。

与持续性低氧（sustained hypoxia, SH）相比，IH对肿瘤生物学行为具有更独特且显著的影响。IH可通过动态调控HIF降解、增强氧化应激、诱导免疫抑制微环境等机制展现出更强的促肿瘤效应。IH导致的氧浓度波动使肿瘤周边形成动态低氧梯度，靠近血管的细胞经历周期性氧分压变化，而肿瘤核心区域则维持相对稳定的低氧状态^[23,24]^。这种空间异质性可通过激活多条信号通路促进肿瘤进展。Karoor等^[25]^研究发现周期性低氧可显著增加小鼠肺转移发生率，HIF-1α可以上调血管内皮生长因子（vascular endothelial growth factor, VEGF）促进肿瘤新生血管形成增多，进一步加剧局部缺氧并形成正反馈环路。HIF-1α还可促进葡萄糖转运蛋白-1（glucose transporter 1, GLUT-1）、乳酸脱氢酶A（lactate dehydrogenase-A, LDH-A）等糖酵解相关酶表达上调，诱导乳酸积累并形成酸性微环境，从而抑制效应T细胞活性并促进Tregs扩增^[26]^。IH对肿瘤免疫微环境的调控还体现在HIF-1α信号轴驱动TAMs向M2型极化，促进髓源性抑制性细胞募集，同时抑制自然杀伤（natural killer cell, NK）细胞细胞毒作用及CD8^+^ T细胞增殖功能。实验证据^[27]^表明IH暴露可下调TAMs中干扰素反应蛋白IFIT1、IFIT3及抗原呈递分子TAP1、TAP2的表达，M1型标志物CD86表达减少和M2型标志物CD206表达增加，整体呈现促肿瘤极化转变。HIF-1α还可通过诱导免疫检查点分子[如程序性死亡配体1（programmed death ligand-1, PD-L1）]表达上调，直接介导肿瘤细胞免疫逃逸。

OSA导致的反复缺氧-复氧循环表现为低氧期抑制HIF-1α降解，复氧期则加速其清除，形成动态循环调控模式^[28]^。这种周期性激活模式与SH诱导的持续性HIF表达显著不同。乳腺癌模型研究文献表明急性低氧更易上调HIF-1α，而慢性低氧则更倾向于稳定HIF-2α，这也解释了不同低氧形态对化疗耐药性影响存在差异的原因。而在黑色素瘤转移模型中，IH诱导的氧化应激与炎症反应协同增强HIF-1α依赖的基质金属蛋白酶（matrix metallopoateinase, MMP）-2和MMP-9及VEGF表达，从而促进肿瘤细胞侵袭与血管生成^[29]^。以上结果提示靶向HIF-1α通路可能成为改善OSA合并肺癌患者预后的潜在干预策略。

### 2.2 炎症与免疫调节

OSA所致的IH状态一方面直接重塑肿瘤微环境，另一方面诱发系统性炎症与免疫失衡，协同促进肿瘤免疫逃逸。在小鼠黑色素瘤肺转移模型^[29]^中，CIH显著上调肿瘤组织中肿瘤坏死因子-α（tumor necrosis factor-α, TNF-α）和白细胞介素-6（interleukin-6, IL-6）的mRNA水平，并增强核因子-κB（nuclear factor-κB, NF-κB）蛋白表达。该炎症激活状态与肿瘤侵袭性呈正相关，而抗氧化剂可有效下调相关炎症标志物表达，提示氧化应激是IH诱导炎症的关键介导机制。

TAMs的功能重塑也在OSA诱导的炎症和免疫调节过程中发挥重要作用，实验结果表明经IH暴露小鼠肿瘤中分离获得的TAMs可显著增强TC1肺癌细胞系的迁移、穿透及增殖侵袭能力。该促肿瘤作用具有细胞接触依赖性，仅在与巨噬细胞共培养时出现，而肿瘤细胞单独接受IH刺激并未产生明显反应^[23]^。IL-17与转化生长因子-β（transforming growth factor-β, TGF-β）信号轴在IH诱发的免疫调控中呈协同效应。其中IL-17通过促进血管新生直接推动肺肿瘤生长，而TGF-β则兼具抑制免疫监视、驱动血管生成及诱导上皮间质转化等多重促瘤作用^[30]^。在卵巢切除模拟的绝经小鼠模型中，IH与卵巢切除均可独立提升TAMs的M2/M1极化比值，提示二者可能通过相似的巨噬细胞调控途径共同促进肿瘤进展。将模型来源的TAMs与小鼠肺癌细胞共培养时，仅IH暴露组TAMs表现出显著促增殖效应，突出IH对免疫微环境具有特异性的重塑能力^[31]^。上述证据系统阐明了OSA相关IH通过炎症-免疫网络多重靶点重塑肿瘤微环境，为临床干预策略提供了新的分子靶点与理论依据。

### 2.3 氧化应激损伤

OSA所致的IH可以显著促进活性氧（reactive oxygen species, ROS）的过度生成，破坏细胞氧化还原稳态。在小鼠肺癌细胞系中，经IH处理后ROS水平明显升高，较常氧条件更为显著。持续的氧化应激状态下产生的ROS直接诱导DNA双链断裂与碱基氧化修饰，从而造成基因组不稳定及突变累积。同时可以激活NOX家族成员，进一步增强氧化损伤效应^[32]^。在NSCLC中NOX4高表达与肿瘤恶性生物学行为有明确相关性。抑制NOX4活性不仅可降低细胞ROS水平，还能显著下调MMP-9表达，以上结果提示氧化应激可能通过调控细胞外基质降解促进肿瘤侵袭。黑色素瘤肺转移模型的实验结果也支持这一观点，OSA所致IH通过ROS诱导氧化应激反应，而抗氧化剂Tempol可有效阻断该过程，该结果提示氧化应激可能是IH促进肿瘤转移的关键机制之一^[19]^。

Bach1是氧化应激损伤中的关键转录调控因子，可以促进多种肿瘤的发生发展，有研究已证实Bach1的高表达与肺癌患者不良预后及转移进展风险显著相关。IH暴露状态可以显著诱导肺部肿瘤组织中的Bach1表达上调，同时抑制抗氧化酶基因转录以进一步加重氧化应激，形成促肿瘤增殖、侵袭、耐药等恶性循环。此外，IH条件下ROS的过量蓄积可显著抑制NK细胞的抗肿瘤作用，削弱机体免疫监视功能。

OSA患者的间歇性缺氧-再氧合循环所生成的ROS具有明确清晰的双重致癌作用，其既可以直接造成DNA损伤，又可以抑制DNA损伤修复过程，从而促使突变累积并加剧基因组的不稳定性^[33]^。这种持续性的DNA损伤状态可能有利于激活原癌基因或失活抑癌基因，从而促进细胞恶性转化。而且研究^[24]^还发现OSA患者的氧化应激标志物水平与肺癌风险呈显著正相关，因此氧化应激有可能是两者关联的核心机制。

### 2.4 代谢紊乱作用

OSA引发的IH可显著破坏机体代谢稳态。在IH条件下，小鼠肺癌细胞系出现明显的代谢表型重塑，其中糖酵解途径激活最为突出。IH环境促进肿瘤细胞向有氧糖酵解代谢转变，该代谢优势既为细胞快速增殖提供能量与生物合成底物，也直接参与调控肿瘤发生、进展及转移^[26]^。IH相关代谢异常与氧化应激反应形成正反馈环路，IH诱导的ROS累积增强糖酵解活性，而糖酵解上调又进一步促进ROS生成，最终形成恶性循环。

IH诱导的代谢紊乱可通过多条关键信号通路调控肿瘤细胞增殖、侵袭和免疫逃逸等生物学行为。IH暴露的小鼠细胞中NADPH氧化酶家族成员NOX2与NOX4的表达显著上调，二者既是氧化应激反应的重要执行者，也直接参与细胞能量代谢调控网络。蛋白质印迹实验结果表明IH处理组较常氧组的VEGF表达显著上调，促进新生肿瘤血管生成，为肿瘤细胞代谢重塑提供持续的营养和氧供支持。代谢重编程过程中生成的乳酸不断在肿瘤微环境中堆积，形成的酸性微环境不仅抑制效应T细胞的杀伤功能，还促进Tregs扩增，从而推动免疫抑制性肿瘤微环境的形成。上述免疫与代谢重塑过程与肿瘤免疫逃逸机制密切相关，这也可能构成OSA患者肺癌进展加速的关键机制。

## 3 治疗策略

OSA与肺癌的共病状态对临床治疗决策提出了特殊挑战。因此在临床诊疗中这类患者的治疗方案宜采用多学科协作的治疗模式，充分整合呼吸科、肿瘤科和睡眠医学等科室的专业意见。近年来众多临床证据已证实OSA症状的有效缓解不仅能够提升肺癌患者围手术期的安全性，也能改善其对放化疗的耐受性，故而为实现治疗协同效应必须要对患者的AHI、肿瘤分子特征以及心肺功能储备等多项指标予以系统、个性化的评估。此外，低氧状态与化疗耐药性、炎症调控及性别差异等临床重要因素的关系也为个性化治疗决策提供了循证医学依据。

### 3.1 持续气道正压通气（continuous positive airway pressure, CPAP）治疗

OSA患者通常采用CPAP作为首选治疗手段，该方案在肺癌患者中的潜在临床价值逐渐受到关注。CPAP能够维持整个呼吸周期内气道持续开放，从而有效缓解OSA患者的夜间低氧状态，并可能改善肺癌患者的临床结局。对确诊为重度OSA的患者接受CPAP治疗前后的外周血淋巴细胞进行转录组测序，结果表明有效的CPAP治疗可以使转录图谱发生改变，许多肿瘤信号通路中关键基因的表达量明显下调^[34]^。一项黑色素瘤的前瞻性多中心研究^[35]^表明未经治疗的中重度OSA是黑色素瘤患者不良预后的独立危险因素，而接受CPAP治疗可以改善患者预后。尽管目前尚缺乏CPAP干预OSA合并肺癌患者并直接改善生存结局的前瞻性临床研究证据，但现有研究已从分子及临床层面提供了间接支持。CPAP治疗可通过纠正IH、减轻氧化应激及炎症反应，从而影响肿瘤相关信号通路的表达。此外，来自黑色素瘤等实体肿瘤的研究文献表明：未治疗的中重度OSA与不良预后有关，而规范CPAP治疗可能带来生存获益。因此，在临床实践中对于合并中重度OSA的肺癌患者，仍建议积极进行CPAP干预，并作为综合抗肿瘤治疗的重要辅助手段之一。

临床研究发现CPAP治疗在血压管理方面具有明确的降压效果。规范CPAP治疗可使患者24 h平均血压降低约3.4/3.3 mmHg。该降压效应对合并心血管疾病的肺癌患者尤为关键，有助于提高其对抗肿瘤治疗的耐受性与安全性。此外，CPAP治疗可能通过改善糖代谢为肺癌患者带来附加获益，现有证据^[36]^表明CPAP能够降低糖尿病患者的夜间血糖水平，这或与缓解IH诱导的胰岛素抵抗有关。OSA相关IH可诱导TNF-α、IL-6和C-反应蛋白（C-reactive protein, CRP）等多种促炎因子持续高表达，从而形成慢性炎症状态，为肿瘤进展提供有利的微环境。有荟萃分析^[36]^显示CPAP治疗可显著降低OSA患者的系统性炎症标志物水平，该效应可能对抑制肺癌的发生与发展具有潜在意义。

临床实践中CPAP治疗当前仍面临诸多挑战，其中患者的低依从性是最突出、最棘手的问题。患者对CPAP治疗普遍存在不耐受，主要是由于设备舒适度不足、面罩贴合度差以及初始治疗阶段引发的不适感所引起。对于OSA合并肺癌患者而言这一问题更为严峻，呼吸困难、剧烈咳嗽等肿瘤相关症状也可能进一步降低其治疗依从性。临床证据表明CPAP可有效改善日间嗜睡状态、稳定血压，但是患者依从性普遍较差，故严重限制了其潜在的临床价值。不同人群对CPAP治疗的反应也存在明显异质性，年轻患者及女性患者对CPAP治疗的依从性更好，而且研究还发现女性患者从CPAP治疗中可以获得更显著的生存获益，这与其较高的治疗依从性密切相关。上述性别差异提示未来优化治疗策略时需要充分审慎地考虑人口学因素的影响。丹麦的一项研究^[37]^提供了有力的证据支持，研究结果表明接受CPAP治疗的OSA患者全因死亡率显著降低（HR=0.62），为CPAP的长期生存获益提供了直接的数据支持。但是需要注意的是现有研究在CPAP治疗依从性评价方面仍有较多不足。多数研究未采用统一、标准化的依从性测量工具，故不同研究所得结果的可比性受到限制。此外，关于神经认知功能与CPAP依从性关系的研究尚不充分，尽管现已有证据^[36]^提示OSA相关认知损害本身也可能影响治疗的持续性。因此在今后研究中应开发更精准可靠的依从性评估体系，继而系统考察影响患者长期治疗坚持度的心理与行为学因素。

### 3.2 药物治疗

OSA与肺癌治疗效果的关系目前尚未完全阐明，现有研究主要聚焦于OSA形成的IH状态对肿瘤免疫微环境的影响。低氧环境还可以增强肿瘤细胞对化疗药物的耐药性，此前研究文献发现在低氧条件下肿瘤细胞可以激活自噬途径抵抗铂类药物所诱导的凋亡，这一机制可能也解释了OSA患者反复夜间低氧导致化疗疗效减弱的现象。OSA可能还通过系统性炎症、免疫功能紊乱等机制来影响治疗效果，实验结果表明IH和睡眠片段化能够重塑肿瘤微环境，促进肿瘤进展。这种微环境改变可以调节免疫细胞功能，从而干扰免疫检查点抑制剂的作用，这也为理解OSA对免疫治疗疗效的影响提供了理论基础。

OSA与肺癌联合治疗的临床研究仍处于探索阶段，流行病学数据揭示约半数肺癌患者合并存在中至重度OSA，这一高共病率凸显了临床实践中开展协同管理的必要性。研究证实OSA与免疫逃逸、新生血管生成及肿瘤侵袭等生物学行为有关，可以引起乳糖凝集素-9（galectin-9, Gal-9）、T细胞免疫球蛋白黏蛋白3（T-cell immunoglobulin and mucin-domain containing-3, TIM-3）等生物标志物水平升高^[38]^。TIM-3及其配体Gal-9是调节肿瘤免疫的关键分子，与PD-L1/程序性死亡受体1（programmed death protein-1, PD-1）信号通路相似，Gal-9与T细胞膜表面的TIM-3受体结合，激活细胞凋亡信号通路，发挥促T细胞耗竭作用。Gal-9/TIM-3信号轴与细胞毒性T细胞的局部浸润和增殖呈负相关，协同形成免疫抑制微环境，发挥免疫逃逸作用^[39]^。研究^[40]^发现血浆中可溶性Gal-9和TIM-3可作为OSA合并肺癌患者的预后标志物，在睡眠呼吸暂停的肺癌和黑色素瘤患者队列中，血浆中可溶性Gal-9和TIM-3浓度升高与肺癌患者的肿瘤体积呈显著正相关，并与肺癌患者的不良预后相关。IH诱导的Gal-9和TIM-3水平升高抑制T细胞功能协助免疫逃逸，亦可增加肿瘤微环境中的肿瘤侵袭性。因此，将缺氧与免疫检查点通路相结合有可能是今后免疫治疗探索的新方向。PD-1和TIM-3联合抑制可发挥协同抗肿瘤免疫治疗作用，TIM-3靶向药物开发在骨髓增生异常综合征和急性髓系白血病中取得初步成效，尽管目前在肺癌中的研究仍有限，基于OSA形成乏氧状态对TIM-3/Gal-9等免疫检查点通路的影响，有理由相信未来TIM-3靶点相关的药物临床试验可能为OSA合并肺癌患者带来新的突破^[41]^。

肥胖是OSA的一个重要的驱动因素，肥胖症患者会加重上呼吸道塌陷，并在睡眠通气过程中需要对抗更高水平脂肪组织的阻力。肥胖症的治疗近10年内发生了革命性的变化，以司美格鲁肽为代表的胰高血糖素样肽-1（glucagon-like peptide-1, GLP-1）受体激动剂成为该治疗领域的焦点^[42]^。GLP-1受体激动剂理论上可以通过控制体重，减少神经肌肉负荷来帮助预防睡眠期间的气道塌陷。一项为期52周的III期临床试验^[43]^研究了替尔泊肽对OSA的疗效，受试者为中重度OSA合并肥胖的患者，1:1随机分组分别接受替尔泊肽和安慰剂联合CPAP治疗，结果表明替尔泊肽组的平均AHI降低幅度（-29.3/h, 95%CI: -33.2--25.4/h）显著高于安慰剂组（-5.5/h, 95%CI: -9.9--1.2/h, P<0.001）。以上结果表明替尔泊肽联合CPAP治疗可以显著改善OSA的症状。Li等^[44]^研究发现非肥胖的OSA患者使用GLP-1受体激动剂后，AHI仍能获得具有临床意义的改善，这提示GLP-1受体激动剂对OSA的疗效可能还包含与体重减轻之外的机制。GLP-1受体可以在中枢神经系统和呼吸控制相关区域表达，这些受体的激活可能影响呼吸模式，GLP-1受体激动剂有可能通过影响呼吸模式发挥治疗作用。临床前研究文献发现利拉鲁肽在体外实验中不会影响小鼠肺癌细胞系的增殖，而在Kirsten大鼠肉瘤病毒癌基因同源物（Kirsten rat sarcoma viral oncogene homolog, KRAS）突变的小鼠肺癌模型中，利拉鲁肽可以显著抑制饮食诱导肥胖小鼠体内的肿瘤生长，但是对正常体重的小鼠无作用。机制研究表明利拉鲁肽可以改变促癌基因表达，同时可以影响肿瘤浸润淋巴细胞的亚群数量和功能变化，从而增强小鼠的抗肿瘤免疫作用。另在一项回顾性队列研究^[45]^中，接受手术治疗的早期NSCLC患者术后接受GLP-1受体激动剂可以延长无复发生存期，联合免疫抑制剂治疗的亚组可以获得最大的无进展生存期和总生存期获益。这与此前的一项关于司美格鲁肽作用于乳腺癌的研究^[46]^结果一致，司美格鲁肽可以增强T细胞杀伤效应，促进树突状细胞成熟，减缓乳腺癌的增殖和扩散。其他研究^[47]^也发现GLP-1受体激动剂在肝细胞癌中也有抗肿瘤作用，利拉鲁肽可以激活NK细胞介导的免疫应答发挥抗肿瘤作用。GLP-1受体激动剂在改善代谢紊乱、减轻体重及调控炎症反应方面具有潜在作用，理论上可能通过改善OSA相关病理状态间接影响肿瘤微环境。然而，目前其在OSA合并肺癌人群中的研究仍局限于基础和探索阶段，缺乏直接临床证据支持，未来仍需开展前瞻性临床研究加以验证。

总而言之，OSA与肺癌的治疗不应彼此独立，而应该采取多学科协同管理策略。手术、化疗、放疗及免疫治疗等规范的抗肿瘤治疗仍是肺癌的核心治疗手段，利用CPAP、体重管理等手段对OSA进行积极干预有助于改善低氧状态、减轻全身炎症反应，从而提高患者对抗肿瘤治疗的耐受性及依从性。因此，在临床实践中肺癌患者应加强对OSA的筛查与长期管理，以期通过提高CPAP治疗依从性及优化综合诊疗方案最大程度改善肺癌患者预后。

## 4 总结与展望

近年来OSA与肺癌相关的研究取得显著进展，证实OSA的严重程度与肺癌发生风险呈剂量依赖关系。尽管多项研究发现OSA与肺癌存在临床关联，但高质量循证医学证据仍不充分。因此今后仍需开展大型、多中心、前瞻性研究，以系统评估OSA治疗对肺癌患者长期结局的影响。

## References

[1] Chang JL, Goldberg AN, Alt JA, et al. International consensus statement on obstructive sleep apnea. Int Forum Allergy Rhinol, 2023, 13(7): 1061-1482. doi: 10.1002/alr.23079 36068685 PMC10359192

[2] Gleeson M, McNicholas WT. Bidirectional relationships of comorbidity with obstructive sleep apnoea. Eur Respir Rev, 2022, 31(164): 210-256. doi: 10.1183/16000617.0256-2021 PMC9488957 35508332

[3] Cheong AJY, Tan BKJ, Teo YH, et al. Obstructive sleep apnea and lung cancer: A systematic review and meta-analysis. Ann Am Thorac Soc, 2022, 19(3): 469-475. doi: 10.1513/AnnalsATS.202108-960OC 34792438

[4] Campos-Rodriguez F, Martinez-Garcia MA, Martinez M, et al. Association between obstructive sleep apnea and cancer incidence in a large multicenter Spanish cohort. Am J Respir Crit Care Med, 2013, 187(1): 99-105. doi: 10.1164/rccm.201209-1671OC 23155146

[5] Cabezas E, Perez-Warnisher MT, Troncoso MF, et al. Sleep disordered breathing is highly prevalent in patients with lung cancer: Results of the Sleep Apnea in Lung Cancer Study. Respiration, 2019, 97(2): 119-124. doi: 10.1159/000492273 30261487

[6] Cho J, Jo S. Association of obstructive sleep apnea with risk of lung cancer: A nationwide cohort study in Korea. Sci Rep, 2024, 14(1): 12394. doi: 10.1038/s41598-024-63238-x 38811831 PMC11137051

[7] Huang T, Lin BM, Stampfer MJ, et al. Associations of self-reported obstructive sleep apnea with total and site-specific cancer risk in older women: A prospective study. Sleep, 2021, 44(3): 12394. doi: 10.1093/sleep/zsaa198 PMC7953220 33015707

[8] Kendzerska T, Povitz M, Leung RS, et al. Obstructive sleep apnea and incident cancer: A large retrospective multicenter clinical cohort study. Cancer Epidemiol Biomarkers Prev, 2021, 30(2): 295-304. doi: 10.1158/1055-9965.EPI-20-0975 33268490 PMC7867627

[9] Marshall NS, Wong KK, Cullen SR, et al. Sleep apnea and 20-year follow-up for all-cause mortality, stroke, and cancer incidence and mortality in the Busselton Health Study cohort. J Clin Sleep Med, 2014, 10(4): 355-362. doi: 10.5664/jcsm.3600 24733978 PMC3960375

[10] Justeau G, Gerves-Pinquie C, Le Vaillant M, et al. Association between nocturnal hypoxemia and cancer incidence in patients investigated for OSA: Data from a large multicenter French cohort. Chest, 2020, 158(6): 2610-2620. doi: 10.1016/j.chest.2020.06.055 32629036

[11] Gozal D, Farre R, Nieto FJ. Obstructive sleep apnea and cancer: Epidemiologic links and theoretical biological constructs. Sleep Med Rev, 2016, 27: 43-55. doi: 10.1016/j.smrv.2015.05.006 26447849 PMC5374514

[12] Wu D, Zhao Z, Chen C, et al. Impact of obstructive sleep apnea on cancer risk: A systematic review and meta-analysis. Sleep Breath, 2023, 27(3): 843-852. doi: 10.1007/s11325-022-02695-y 36129602

[13] Qi P, Qi B, Ding Y, et al. Implications of obstructive sleep apnea in lung adenocarcinoma: A valuable omission in cancer prognosis and immunotherapy. Sleep Med, 2023, 107: 268-280. doi: 10.1016/j.sleep.2023.05.013 37263079

[14] Lee H, Kim HH, Kim KY, et al. Associations among sleep-disordered breathing, sleep quality, and lung cancer in Korean patients. Sleep Breath, 2023, 27(4): 1619-1628. doi: 10.1007/s11325-022-02750-8 36434375

[15] Liu W, Zhou L, Zhao D, et al. Development and validation of a prognostic nomogram in lung cancer with obstructive sleep apnea syndrome. Front Med (Lausanne), 2022, 9: 810907. doi: 10.3389/fmed.2022.810907 35372417 PMC8971712

[16] Martinez-Garcia MA, Campos-Rodriguez F, Duran-Cantolla J, et al. Obstructive sleep apnea is associated with cancer mortality in younger patients. Sleep Med, 2014, 15(7): 742-748. doi: 10.1016/j.sleep.2014.01.020 24907033

[17] Liu W, Luo M, Fang YY, et al. Relationship between occurrence and progression of lung cancer and nocturnal intermittent hypoxia, apnea and daytime sleepiness. Curr Med Sci, 2019, 39(4): 568-575. doi: 10.1007/s11596-019-2075-6 31346992

[18] Huang HY, Lin SW, Chuang LP, et al. Severe OSA associated with higher risk of mortality in stage III and IV lung cancer. J Clin Sleep Med, 2020, 16(7): 1091-1098. doi: 10.5664/jcsm.8432 32209219 PMC7954049

[19] Li L, Lu J, Xue W, et al. Target of obstructive sleep apnea syndrome merge lung cancer: Based on big data platform. Oncotarget, 2017, 8(13): 21567-21578. doi: 10.18632/oncotarget.15372 28423489 PMC5400607

[20] Hao S, Li F, Jiang P, et al. Effect of chronic intermittent hypoxia-induced HIF-1alpha/ATAD2 expression on lung cancer stemness. Cell Mol Biol Lett, 2022, 27(1): 44. doi: 10.1186/s11658-022-00345-5 35672694 PMC9172155

[21] Marhuenda E, Campillo N, Gabasa M, et al. Effects of sustained and intermittent hypoxia on human lung cancer cells. Am J Respir Cell Mol Biol, 2019, 61(4): 540-544. doi: 10.1165/rcmb.2018-0412LE 31573339

[22] Torres M, Campillo N, Nonaka PN, et al. Aging reduces intermittent hypoxia-induced lung carcinoma growth in a mouse model of sleep apnea. Am J Respir Crit Care Med, 2018, 198(9): 1234-1236. doi: 10.1164/rccm.201805-0892LE PMC6221575 30016601

[23] Hunyor I, Cook KM. Models of intermittent hypoxia and obstructive sleep apnea: Molecular pathways and their contribution to cancer. Am J Physiol Regul Integr Comp Physiol, 2018, 315(4): R669-R687. doi: 10.1152/ajpregu.00036.2018 29995459

[24] Gozal D, Farre R, Nieto FJ. Putative links between sleep apnea and cancer: From hypotheses to evolving evidence. Chest, 2015, 148(5): 1140-1147. doi: 10.1378/chest.15-0634 26020135 PMC4631033

[25] Karoor V, Le M, Merrick D, et al. Alveolar hypoxia promotes murine lung tumor growth through a VEGFR-2/EGFR-dependent mechanism. Cancer Prev Res (Phila), 2012, 5(8): 1061-1071. doi: 10.1158/1940-6207.CAPR-12-0069-T 22700853 PMC4169262

[26] Chen A, Sceneay J, Godde N, et al. Intermittent hypoxia induces a metastatic phenotype in breast cancer. Oncogene, 2018, 37(31): 4214-4225. doi: 10.1038/s41388-018-0259-3 29713057

[27] Almendros I, Wang Y, Becker L, et al. Intermittent hypoxia-induced changes in tumor-associated macrophages and tumor malignancy in a mouse model of sleep apnea. Am J Respir Crit Care Med, 2014, 189(5): 593-601. doi: 10.1164/rccm.201310-1830OC PMC3977714 24471484

[28] Kang HS, Kwon HY, Kim IK, et al. Intermittent hypoxia exacerbates tumor progression in a mouse model of lung cancer. Sci Rep, 2020, 10(1): 1854. doi: 10.1038/s41598-020-58906-7 PMC7002457 32024881

[29] Li L, Ren F, Qi C, et al. Intermittent hypoxia promotes melanoma lung metastasis via oxidative stress and inflammation responses in a mouse model of obstructive sleep apnea. Respir Res, 2018, 19(1): 28. doi: 10.1186/s12931-018-0727-x 29433520 PMC5809953

[30] Chen LD, Lin L, Chen JZ, et al. Identification of key genes in chronic intermittent hypoxia-induced lung cancer progression based on transcriptome sequencing. BMC Cancer, 2024, 24(1): 41. doi: 10.1186/s12885-023-11785-3 38183079 PMC10770984

[31] Torres M, Martinez-Garcia MA, Campos-Rodriguez F, et al. Lung cancer aggressiveness in an intermittent hypoxia murine model of postmenopausal sleep apnea. Menopause, 2020, 27(6): 706-713. doi: 10.1097/GME.0000000000001526 32108736

[32] Zheng Y, Yang S, Si J, et al. Shashen-Maidong decoction inhibited cancer growth under intermittent hypoxia conditions by suppressing oxidative stress and inflammation. J Ethnopharmacol, 2022, 299: 115654. doi: 10.1016/j.jep.2022.115654 36058477

[33] Sunnetcioglu A, Alp HH, Sertogullarindan B, et al. Evaluation of oxidative damage and antioxidant mechanisms in COPD, lung cancer, and obstructive sleep apnea syndrome. Respir Care, 2016, 61(2): 205-211. doi: 10.4187/respcare.04209 26604330

[34] Gharib SA, Seiger AN, Hayes AL, et al. Treatment of obstructive sleep apnea alters cancer-associated transcriptional signatures in circulating leukocytes. Sleep, 2014, 37(4): 709-714, 714A-714T. doi: 10.5665/sleep.3574 24688164 PMC3954174

[35] Gomez-Olivas JD, Campos-Rodriguez F, Nagore E, et al. Role of sleep apnea and long-term CPAP treatment in the prognosis of patients with melanoma: A prospective multicenter study of 443 patients. Chest, 2023, 164(6): 1551-1559. doi: 10.1016/j.chest.2023.06.012 37348828

[36] Prisant LM, Dillard TA, Blanchard AR. Obstructive sleep apnea syndrome. J Clin Hypertens (Greenwich), 2006, 8(10): 746-750. doi: 10.1111/j.1524-6175.2006.888139.x PMC8109598 17028491

[37] Jennum P, Tonnesen P, Ibsen R, et al. All-cause mortality from obstructive sleep apnea in male and female patients with and without continuous positive airway pressure treatment: A registry study with 10 years of follow-up. Nat Sci Sleep, 2015, 7: 43-50. doi: 10.2147/NSS.S75166 25914563 PMC4399513

[38] Cubillos-Zapata C, Troncoso-Acevedo F, Diaz-Garcia E, et al. Sleep apnoea increases biomarkers of immune evasion, lymphangiogenesis and tumour cell aggressiveness in high-risk patients and those with established lung cancer. ERJ Open Res, 2024, 10(1): 00777-2023. doi: 10.1183/23120541.00777-2023 38375428 PMC10875459

[39] Hill CN, Maita G, Cabrolier C, et al. Galectin-9 and Tim-3 in gastric cancer: A checkpoint axis driving T cell exhaustion and Treg-mediated immunosuppression independently of anti-PD-1 blockade. Front Immunol, 2025, 16: 1600792. doi: 10.3389/fimmu.2025.1600792 40666515 PMC12259562

[40] Diaz-Garcia E, Alfaro E, Perez-Moreno P, et al. Immune checkpoint biomarkers galectin-9 and TIM-3 predict melanoma and lung cancer mortality in obstructive sleep apnoea. Arch Bronconeumol, 2025, 61(12): 735-748. doi: 10.1016/j.arbres.2025.03.018 40287376

[41] Gomes de Morais AL, Cerda S, de Miguel M. New checkpoint inhibitors on the road: Targeting TIM-3 in solid tumors. Curr Oncol Rep, 2022, 24(5): 651-658. doi: 10.1007/s11912-022-01218-y 35218498

[42] Wilding JPH, Batterham RL, Calanna S, et al. Once-weekly semaglutide in adults with overweight or obesity. N Engl J Med, 2021, 384(11): 989-1002. doi: 10.1056/NEJMoa2032183 33567185

[43] Malhotra A, Grunstein RR, Fietze I, et al. Tirzepatide for the treatment of obstructive sleep apnea and obesity. N Engl J Med, 2024, 391(13): 1193-1205. doi: 10.1056/NEJMoa2404881 38912654 PMC11598664

[44] Li M, Lin H, Yang Q, et al. Glucagon-like peptide-1 receptor agonists for the treatment of obstructive sleep apnea: A meta-analysis. Sleep, 2025, 48(4): zsae280. doi: 10.1093/sleep/zsae280 39626095

[45] Pachimatla AG, Fitzgerald B, Ogidigo J, et al. Glucagon-like peptide-1 receptor agonism improves lung cancer outcomes and tumor growth control. JCI Insight, 2025, 10(19): e195484. doi: 10.1172/jci.insight.195484 40857107 PMC12513478

[46] Stanisavljevic I, Pavlovic S, Simovic Markovic B, et al. Semaglutide decelerates the growth and progression of breast cancer by enhancing the acquired antitumor immunity. Biomed Pharmacother, 2024, 181: 117668. doi: 10.1016/j.biopha.2024.117668 39536536

[47] Chen D, Liang H, Huang L, et al. Liraglutide enhances the effect of checkpoint blockade in lung and liver cancers through the inhibition of neutrophil extracellular traps. FEBS Open Bio, 2024, 14(8): 1365-1377. doi: 10.1002/2211-5463.13499 PMC11301266 36271684

